# Simultaneous rupture of the anterior cruciate ligament and patellar tendon: report of a case and review of the literature

**DOI:** 10.1016/j.ijscr.2025.111376

**Published:** 2025-04-25

**Authors:** Mansi Zied, Ben Salah Jihed, Guedhami Hedhili, Souid Abderrahmen, Jlalia Zied, Soui Slah

**Affiliations:** a Department of Orthopedic Surgery, IBN EL JAZZAR University Hospital, Kairouan, Tunisia; b Department of Orthopedic Surgery, Hospital of Gafsa, Gafsa, Tunisia; c Orthopedic pediatric department, Kassab institute of orthopedic surgery, Tunisia; d Department of anesthesia, IBN EL JAZZAR University Hospital, Kairouan, Tunisia

**Keywords:** Rupture, Anterior cruciate ligament (ACL), Patellar tendon, Ligamentoplasty

## Abstract

**Introduction and importance:**

Simultaneous rupture of the anterior cruciate ligament (ACL) and patellar tendon is a rare and complex injury, often resulting from high-energy trauma. This dual injury presents significant challenges in diagnosis and treatment.

**Case presentation:**

This is a 29-year-old patient who sustained simultaneous ruptures of the ACL and patellar tendon following right knee trauma. A single-stage surgical treatment was performed, which involved ACL ligament reconstruction using a tendon graft from the contralateral knee and an end-to-end suture of the patellar tendon reinforced with a figure-8 plasty from the ipsilateral knee. The functional results were good: 140 degrees of flexion and full extension with stable support. Clinical evaluation included IKDC, Lysholm, and KOOS scores. The patient returned to work after three months.

**Discussion:**

Such combined injuries are rare, and their diagnosis often requires thorough clinical assessment and imaging, especially MRI. Treatment protocols remain debated, with differing opinions on one-stage versus two-stage surgeries. However, early diagnosis and repair of the patellar tendon have been shown to provide the best outcomes. Our proposed technique aims for optimal functional results, emphasizing early intervention, precise repair, and personalized rehabilitation: analgesic physiotherapy, active extension and strengthening work.

**Conclusion:**

In conclusion, suspicion of simultaneous ACL and patellar tendon rupture should arise in high velocity acute knee trauma with ligamentous laxity and significant hemarthrosis. Prompt diagnosis is the key of management. While a well-established treatment protocol is lacking, the general consensus is that prompt intervention is crucial for the patellar tendon.

## Highlights

• Simultaneous rupture of the anterior cruciate ligament (ACL) and patellar tendon is a rare and complex injury
• Such combined injuries are rare, and their diagnosis often requires thorough clinical assessment and imaging, especially MRI
• Reconstruction of chronic patellar tendon ruptures using the ipsilateral semitendinosus tendon mimicking the native tendon, appears to be an anatomical method

## 1 Introduction

The simultaneous rupture of the anterior cruciate ligament (ACL) and patellar tendon is an uncommon and complex injury that typically results from high-impact trauma. In the literature, we have only been able to identify a dozen similar cases. This type of injury, often seen in events such as motor vehicle accidents, sports-related incidents, or falls from significant heights, presents a unique challenge for both diagnosis and treatment. The severity of the injury requires a comprehensive understanding of the biomechanics of the knee, as well as advanced surgical techniques to ensure optimal outcomes. Timely diagnosis and appropriate intervention are crucial, as the involvement of both key stabilizing structures of the knee can severely impact function, mobility, and long-term joint health. Addressing these challenges requires a multidisciplinary approach that integrates clinical expertise, imaging technology, and surgical precision.

## 2 Methods

The work has been reported in line with the SCARE criteria [1].

## 3 Case presentation

**Fig. 1:**
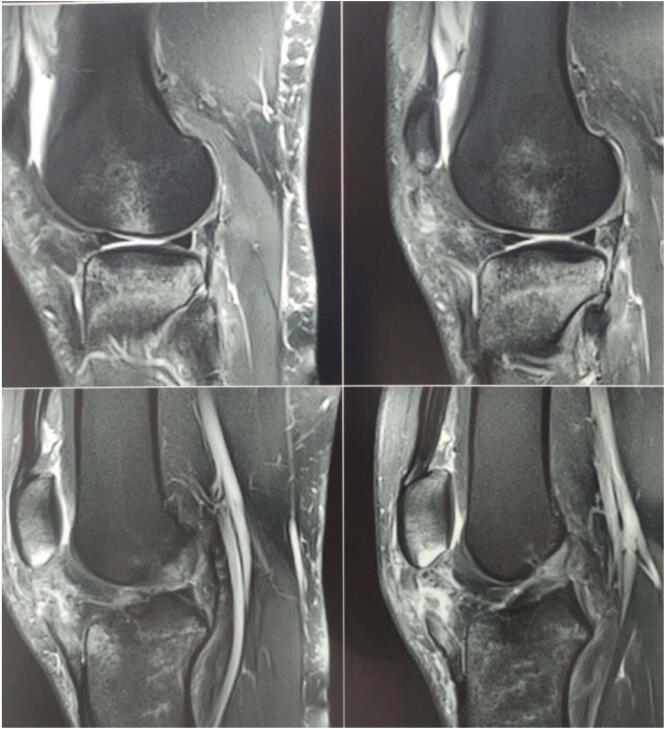
MRI: complete rupture of the patellar tendon and anterior cruciate ligament.

**Fig. 2:**
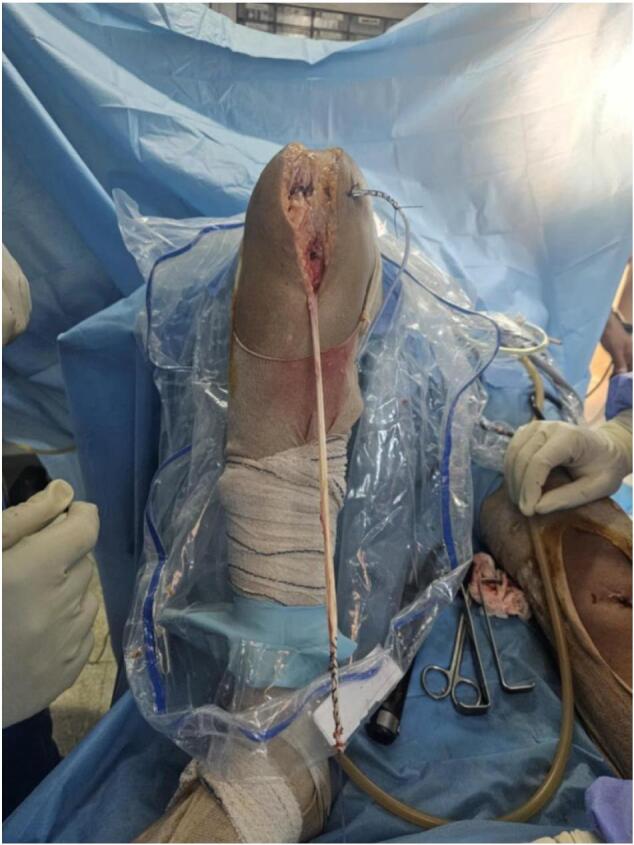
End-to-end sutures with PDS2 and Harvesting of the semitendinosus and gracilis tendons.

**Fig. 3:**
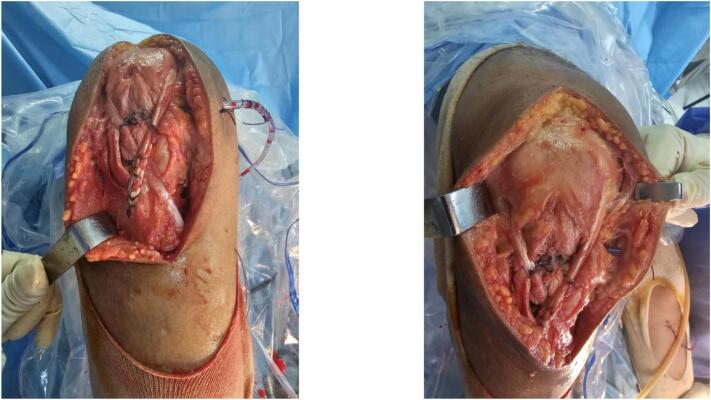
Repair of the patellar tendon reinforcement with a DIDT graft from the ipsilateral knee, passed through a transverse trans-patellar tunnel and secured at the external tibial tuberosity with an interference screw, forming a figure-8 pattern.

**Fig. 4:**
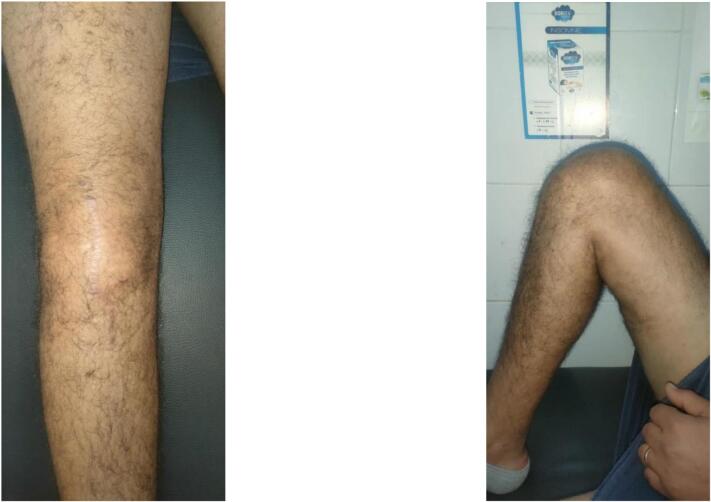
3-month follow-up; full active extension, and flexion at 130°.

We report the case of a 29-year-old patient, without any pre-existing disease, victim of a traffic accident falling from a motorcycle and landing on the right knee with notion of immediate post-traumatic functional impotence and sensation of cracking. Initial management included symptomatic treatment and knee immobilization with a brace. One week later, physical examination revealed increased swelling in the right knee, a deficit in active extension, and an inability to bear weight on the affected leg. Notable findings included a positive patellar shock test, patellar ascension, and a sub-patellar gap. Along with evident damage to the extensor mechanism, the patient exhibited a positive anterior drawer test at 90° of knee flexion, a soft endpoint during the Trillat-Lachmann test, and a positive pivot shift test. Standard X-rays revealed patella alta, and MRI confirmed a complete rupture of both the patellar tendon and anterior cruciate ligament (ACL) (Fig. 1). The patient underwent surgery on day 13 post-trauma. The ACL was repaired using a DIDT (Double-Looped Iliotibial Band) ligamentoplasty graft from the contralateral knee under arthroscopic guidance. Additionally, the patellar tendon was repaired with an end-to-end suture using non-absorbable Nylon 2 thread through an anterior trans-patellar tendon approach. The procedure also involved reinforcement with a DIDT graft from the ipsilateral knee, passed through a transverse trans-patellar tunnel and secured at the external tibial tuberosity with an interference screw, forming a figure-8 pattern (Fig. 2, Fig. 3). The semitendinosus and internal rectus tendons were harvested while preserving their tibial attachments (crow's feet). The patient's postoperative course was uncomplicated. They were immobilized with a knee brace for 4 weeks and initially supported with two canes. Active extension exercises began the day after surgery and continued for the first 15 days. 3 At the 3-month follow-up, the results were satisfactory: a negative Trillat-Lachman test (indicating no instability), no signs of yielding, full active extension, and flexion at 130°. The patient gradually returned to work, though there was mild right quadriceps hypotrophy, which was effectively addressed through 3 months of rehabilitation (Fig. 4). By the 6-month mark, the patient had achieved favourable results: an IKDC score indicating good knee function: absence of symptoms (pain, stiffness, swelling, locking or sliding of the knee), restoring knee function and sports activities; Moreover, both the Lysholm and KOOS scores were 100/100. There was no evidence of quadriceps asymmetry at this stage.

## 4 Discussion

Simultaneous rupture of both the anterior cruciate ligament (ACL) and the patellar ligament is a rare and complex injury, predominantly seen in male patients between the ages of 15 and 30. only a few similar cases have been reported in the litterature. This injury is typically caused by high-energy trauma, such as multi-trauma incidents or severe sports injuries. Several factors increase the risk of such injuries, including repetitive microtrauma, tendinopathies (e.g., jumper’s knee, Sinding-Larsen-Johannson disease), and iatrogenic factors (e.g., corticosteroid injections or previous knee surgeries) [2]. A review of the literature shows only a few documented cases, many of which also involve additional ligament injuries, particularly to the medial collateral ligament (MCL) [3]. In some instances, the patellar ligament rupture was discovered incidentally during surgery for ACL or LCL repairs. Additionally, there were cases where the ACL injury was missed initially and only identified during the surgical repair of the patellar ligament rupture. Previous cases of simultaneous ACL and patellar tendon ruptures have not clearly defined a specific mechanism of injury, as the causes were not well established in Rae and Davies’s [4] case and were inconsistent across other reports. However, in the current case, as well as in at least five out of eight patients with known mechanisms, the injuries occurred during landing or takeoff from a jump without direct impact. This suggests that the mechanism behind simultaneous ruptures may be similar to that of isolated patellar tendon ruptures, where high-stress movements like jumping lead to injury without any direct trauma. Diagnosis of concurrent ACL and patellar ligament ruptures is often delayed due to overlapping clinical symptoms, such as hemarthrosis and significant pain, which are common in other knee injuries [3]. The lack of immediate post-traumatic MRI scans and the similar mechanisms of injury make early detection challenging. However, in the current case, the diagnosis was made promptly through clinical findings, such as the inability to fully extend the knee, subpatellar depression, and a positive Lachman test. Since making a definitive diagnosis based only on history and physical examination can be difficult, radiological investigations are crutial for confirmation. Radiographs can reveal Patella Alta by showing an elevated position of the patella, while MRI scans offer detailed views of the knee's soft tissues and cartilage. These imaging methods contribute to a quicker and more precise diagnosis, helping to exclude other possible conditions and guide effective treatment. 4 Due to the rarity of this injury, there is no standardized treatment protocol [3]. However, early diagnosis and surgical intervention are crucial for the best outcomes. The goal of treatment is restoration of the extensor mechanism, active and passive movements in the knee joint and absence of axial load to restore knee stability. Research indicates that prompt repair of the patellar tendon is essential, as delays beyond six weeks may result in complications such as quadriceps atrophy, patellar ascent, and limited knee flexion [2]. Some experts recommend a two-stage surgical approach [4]: first, an early repair of the patellar ligament to prevent further damage, followed by ACL reconstruction once inflammation subsides and knee range of motion improves. In most cases, end-to-end sutures reinforced with various materials such as synthetic plastics or metal wires are used, sometimes in combination with prosthetic materials like polydioxanone (PDS) [2,3]. Conversely, ACL reconstruction is typically delayed to allow the inflammatory phase to resolve and to avoid complications [4]. A "cold" knee (free from significant edema and inflammation), along with improved range of motion and restored knee extension, are key indicators for minimizing the risk of post-operative arthrofibrosis and Baja patella (abnormally low patella) [4]. However, other studies advocate for single-stage surgery, arguing that the timing of the procedure should be determined by factors such as the availability of an appropriate graft, the patient's tissue condition, and overall health status [4,5]. In the case of our patient, we opted for a combined approach, performing a DIDT type ligamentoplasty for the ACL along with simultaneous repair of the patellar tendon and reinforcement with a DIDT graft from the ipsilateral knee. Importantly, no osteosynthesis with cerclage wire was required. Furthermore, early rehabilitation, including active knee extension exercises and weight bearing support, was initiated on the first day after surgery and by the third post-operative week, the crutches are removed to allow for full weight-bearing. This approach supports the potential for improved functional outcomes and reduced complications compared to a delayed, two-stage procedure. In the cases reported in the literature, the patellar tendon is typically repaired without the use of reinforcement plasty, although it may sometimes be reinforced with cerclage wire. In such instances, autografting the patellar tendon for ACL reconstruction is not feasible. Instead, the recommended approach is to use an autograft from the hamstring tendons, specifically the semitendinosus and gracilis. This choice is preferred due to the ease of harvesting these tendons and their ability to provide a rapid functional recovery of the knee, all while preserving the integrity of the extensor mechanism. Postoperative care typically involves 3 to 6 weeks of immobilization using a knee brace, cast splint, or removable orthosis. Early rehabilitation, beginning with passive mobilization on the first postoperative day, is essential to ensure proper knee function and prevent stiffness. Active rehabilitation can be introduced as the knee stabilizes and strength improves [6]. Failure to identify an ACL injury during the initial trauma evaluation can complicate surgical management and may result in inadequate treatment, leading to long-term functional impairments. In such instances, clinicians must be mindful of the potential medico-legal 5 consequences, as delays in diagnosis and treatment can adversely affect recovery and lead to legal challenges, especially in cases of complex multi-ligament knee injuries [7]. Therefore, early diagnosis, timely intervention, and tailored surgical planning are critical to optimizing functional outcomes in patients with simultaneous ACL and patellar ligament ruptures. From our knowledge, there is no study in the literature documenting this surgical procedure.

## 5 Conclusion

In summary, this case highlights the significance of early identification and timely surgical management in patients with concurrent ACL and patellar tendon ruptures. Indeed, a thorough a careful clinical examination and the early use of an MRI are crucial for initiating appropriate treatment. The use of a single-stage surgical procedure, followed by an early rehabilitation program, contributed to a successful recovery. Due to the uncommon nature of this injury, further investigations are required to develop standardized treatment guidelines and optimize functional results in similar cases.

## CRediT authorship contribution statement

Dr. Mansi Zied: study concept, data collection, interpretation, Supervision, Validation

Dr. Ben salah Jihed: analysis, data collection, writing the paper

Dr. Guedhami Hedhili: analysis and data collection

Dr. Souid Abderrahmen: analysis and data collection

Dr. Jlalia Zied: study concept and data collection

Dr. Soui Slah: interpretation, Validation

## Consent

Written informed consent was obtained from the patient for publication and any accompanying images. A copy of the written consent is available for review by the Editor-in-Chief of this journal on request.

## Ethical approval

Ethical approval for this study (Ethical Committee N°19) was provided by the Ethical Committee of IBN EL JAZZAR Hospital, Kairouan, Tunisia on 10 January 2024.

## Guarantor

Mansi Zied

## Funding

There is no source of funding for our research.

## Declaration of competing interest

The authors confirm that they have no conflicts of interest associated with this publication.

The authors declare that they have no known competing financial interests or personal relationships that could have appeared to influence the work reported in this paper.
